# Molecular Targets for Endometriosis Therapy: Where We Are and Where We Are Going?

**DOI:** 10.22074/ijfs.2019.5736

**Published:** 2019-04-27

**Authors:** Fabio Barra, Antonio Simone Laganà, Jvan Casarin, Fabio Ghezzi, Lorenzo Ferro Desideri, Carolina Scala, Simone Ferrero

**Affiliations:** 1 Academic Unit of Obstetrics and Gynecology, Ospedale Policlinico San Martino, Largo R. Benzi, Genoa, Italy; 2 Department of Neurosciences, Rehabilitation, Ophthalmology, Genetics, Maternal and Child Health (DiNOGMI), University of Genoa, Genoa, Italy; 3 Department of Obstetrics and Gynecology, Filippo Del Ponte Hospital, University of Insubria, Varese, Italy

Endometriosis is a chronic hormone-dependent disease, characterized by the presence of endometrial-like glands and stroma outside the uterine cavity. It could occur in distinct phenotypes: peritoneal superficial lesions, ovarian endometriomas and deep infiltrating endometriosis (DIE) (1), which includes various locations penetrating >5 mm under the peritoneal surface. Prevalence of this disease in women of child-bearing age ranges between 6 and 10% (2), but it may also be diagnosed sometimes in menopause. Endometriosis may be responsible for pain symptoms and infertility, which can severely impact the patient’s quality of life (3). Transvaginal ultrasonography is the non-invasive gold standard technique for diagnosing DIE and ovarian endometriomas (4). Magnetic resonance imaging (MRI) can be helpful when the gynecologist is not experienced about ultrasonographic diagnosis of endometriosis or when the findings of ultrasonography are ambiguous (5). Anyway, confirmation of endometriosis diagnosis is only achieved by histological analysis of endometrial stroma and glands.

Medical therapy is usually the first-line option to treat women affected by endometriosis, aiming to improve patient’s pain symptoms and to prevent disease recurrence after surgery. Indeed, progestins and combined oral contraceptives (COCs) are usually started in patients with suspicion of endometriosis without any surgical diagnosis (6). Currently, the most appropriate therapy is chosen taking into account several factors, such as patients’ age, preference, desire to conceive, intensity and features of pain. Anyway, a long-term regimen is necessary for patients affected by this benign chronic disease, in which, efficacy in improving symptoms has to be balanced with a good tolerability.

Currently available options are not definitely curative for endometriosis, even if women have temporary relief of symptoms. Nevertheless, once the therapy is discontinued, their recurrence happens. Moreover, treatments employed in the clinical practice, with the exception of non-steroidal anti-inflammatory drugs (NSAIDs), are contraceptive, representing a challenge for patients whose want to become pregnant (7). For these reasons, the research of novel alternative active drugs is mandatory.

In this regard, the increasing knowledge of several molecular pathways involved in the genesis of this chronic and progressive disease has pushed forward the investigation of new interesting targets. It is known that implantation, growth and progression of endometriosis are caused by a number of disturbed biological mechanisms including invasion capacity, cell proliferation, apoptosis (8), immune function (9-11) as well as angiogenesis (12).

Research is focalized on finding drugs that specifically target the hormonal and immunological microenvironment of implants, down-regulating endometriotic cells proliferation, enhancing their apoptosis as well as renormalizing their up-regulated mechanisms of invasion and angiogenesis. Over the last 20 years, a wide variety of medical options has been tested: in particular, among experimental hormonal compounds, aromatase inhibitors and gonadotropin realizing hormone (GnRH)-antagonists have been the most studied drug classes in late clinical trials.

Investigation of aromatase inhibitors has greatly been increased over the last decade, considering that important role of aromatase enzyme has been demonstrated in the endometriotic implants. However, majority of data concerning the use of aromatase inhibitors includes a low number of patients receiving them for a limited period of time (maximum 6 months). In addition, frequent presence of drug-related adverse events (such as vasomotor symptoms and musculoskeletal pain) represents an important limitation for their clinical long-term use. Furthermore, it has been reported that increasing serum follicle-stimulating hormone (FSH) levels, by these drugs, may cause development of ovarian cysts (13). Nevertheless, in our opinion, research of alternative formulation of aromatase inhibitors is still appealing. For example, a combination of anastrozole and levonorgestrel in a vaginal ring is under evaluation in a randomized, double-blind phase II trial (NCT02203331). In particular, we deem that this vaginal combination may be advantageous for patients with rectovaginal nodules of endometriosis, considering a local action of this drug in high concentration. Anyway, at the moment, administration of aromatase inhibitors should be reserved only in patients not responding to the conventional therapies in the setting of scientific investigations (14).

Contrary to the form of GnRH-analogs, GnRH-antagonists maintain sufficient circulating levels of estrogens, contributing to avoid vasomotor symptoms as well as loss of bone mineral density (15). After promising findings in the multicenter, randomized, double-blinded Elaris Endometriosis I-II studies (16), long-term oral elagolix was effective in improving dysmenorrhea (overall 46-76% of patients) and chronic pelvic pain (50-76%) due to endometriosis with a good safety-profile. These promising data demonstrate that elagolix (in particular at 150 mg, once daily) might be a potential candidate for the management of patients with endometriosis-associated pain who are not responsive to COCs or progestins without the necessity for add-back therapy (differently from the GnRH-analogs). Anyway, new ongoing multicenter double-blinded phase III studies should confirm these preliminary results on larger populations (NCT03343067, elagolix alone; NCT03213457, elagolix plus NETA and estradiol). Moreover, relugolix (TAK-385), as another GnRH-antagonist (17), is currently under investigation in an international phase III study in comparison with placebo (NCT03204318). In general, we think that for this group of innovative hormonal drugs, randomized trials should be necessary in order yet also to compare GnRH-antagonists with COCs or progestins.

Selective hormonal receptor modulators have variable effects on estrogen and progesterone receptors of different tissues, as their pharmacodynamics activity ranges from pure agonism to a pure antagonism. Anyway, the use of selective estrogen receptor modulators (SERMs) and selective progesterone receptor modulators (SPRMs) is improbable to become a first-line strategy to treat endometriosis due to the unproductive results observed in laboratory and animal studies (18). Firstly, these drugs target the same receptors and have the same therapeutic mechanisms with the available hormonal compounds, including the potential contraceptive effect. More importantly, a randomized controlled phase II trial that evaluated a 6-months therapy with raloxifene was prematurely interrupted, because the women allocated to the SERM group showed worsening pain (19). In fact, it has been proposed that raloxifene, differently from rodents that have an estrous cycle, in human may not be able to prevent ovulation. The subsequent production of ovarian estrogens may be continued and, in some cases, even increased following the action on receptors of this drug, causing a worsening of the symptoms. Regarding SPRMs, the studies on anoprisnil have been stopped for the presence of some cases of endometrial hyperplasia (18). Ulipristal, largely used for preoperatively treating myomas, has been investigated by the Pharmacovigilance Risk Assessment Committee of the European Medicines Agency (EMA) for four recent cases of serious liver injury, and thus, its future testing for endometriosis appears improbable.

Currently, new hormonal drugs acting on steroid sulfatase and 17β-hydroxysteroid dehydrogenase are under early pre-clinical investigation (20). Interestingly, a first dual inhibitor of these two pathways has been developed, but no in vitro experiment or investigation on animals with endometriosis has been reported yet (21). Generally, more information on efficacy and safety in animals is needed (in particular for 17β-hydroxysteroid dehydrogenase inhibitors) before being able to translate these experimental compounds to women affected by endometriosis. Other non-hormonal targets have been preliminary tested for endometriosis. Anyway, few studies on human have been organized for these compounds.

Angiogenesis is an essential process for establishment and development of implants of endometriosis. Several anti-angiogenic agents (anti-angiogenic antibodies or multi-tyrosine kinases and mTor inhibitors) have been tested in rodents, showing good efficacy in reducing growth of endometriotic lesions (22). However, we deem that the pharmacological activity against angiogenesis in nodules of DIE, which tends to have a high content of fibrosis, remains unclear. Furthermore, due to the important drug-related adverse events (associated to the interference with physiologic angiogenesis) their potential translation into human research should be taken with caution. At the moment, almost none of them have been investigated in clinical trials.

A small pilot study reported encouraging findings in terms of efficacy (70% reduction of endometriotic implants size) and safety by administering quinagolide, a dopamine agonist, for treatment of hyperprolactinemic patients with peritoneal endometriosis. It has been reported by the authors that the mechanism of action of this drug may include an inhibitory effect on the angiogenic pathway (showed in their study by the down-regulation of VEGF/VEGFR2 expression). However, no data regarding patients’ symptoms have been reported in this trial (23). Thus, larger, better-designed studies, focalized on clinical efficacy should be organized to confirm its efficacy for endometriosis.

Targeting inflammatory-related pathways in endometriosis appears rational, as it is widely known that the overproduction of prostaglandins, cytokines and other pro-inflammatory mediators characterize endometriotic tissue. Moreover, the action mechanism of non-selective NSAIDs, is largely employed and it is effective for the treatment of pain associated to endometriosis, including inhibition of the pro-inflammatory prostaglandins synthesis at both the COX-1 and COX-2 sites. For this reason, tumor necrosis factor-α (TNF-α) inhibitors, such as etanercept or infliximab commonly administered in clinical practice for treatment of chronic inflammatory diseases, have been investigated for this chronic hormonal disease (18). Contrary to expectations after promising results in animal models, no clinical trials have been carried out in this setting with the exception of infliximab, which did not demonstrate to be enough active in a small clinical study on women (24).

Oxidative stress displays an important role in the development and progression of endometriotic implants (25). The efficacy of several antioxidants for improving endometriosis-associated pain and reducing size of implants has been successfully assessed in animals. In future, it would be interesting to investigate other potential drugs, like statins, metformin and tiazolinediones, which are not expensive and they are largely available. Moreover, according to latest evidences, they may exert both antioxidant and anti-inflammatory proprieties (26). In this regard, a well-designed in vitro study of human endometrial biopsies and 3-D culture in fibrin matrix investigated the effects of lovastatin on proliferation of stromal cells and invasion of the fibrin matrix (27). Interestingly, a concentration-dependent effect of lovostatin was seen on cell growth and angiogenesis in the experimental groups. Particularly, in the presence of 5 and 10 μM of statin, angiogenesis was abolished, and cell proliferation was inhibited. In the presence of 1 μM of lovastatin, angiogenesis was reduced, but cell proliferation was not affected.

Anyway, a small clinical trial has only been organized to test statins in women after conservative surgery in order to prevent recurrence of endometriosis (28). However, further large studies in vivo are required to draw more accurate conclusions on this topic.

Lastly, methylation of progesterone receptor gene may be part of aberrant gene silencing described in endometriosis (29). Thus, demethylation agents as well as histone deacetylase inhibitors were proposed as potential therapeutic options. At the moment, safety profile of these drugs should be deeply weighed before considering eventual clinical application. In particular, among the others, valproic acid seems to be the most promising option, considering the interesting results obtained from a series of patient case with adenomyosis (30). Anyway, no trial on human has been performed for endometriosis and therefore their beneficial effect should still be confirmed.

In conclusion, a high number of new investigational drugs targeting specific biological mechanisms have recently been proposed for treatment of endometriosis. Although the use of new hormonal treatments, such as GnRH-antagonists, is being deeply tested in the last clinical studies appearing relatively near to introduce into clinical practice, the majority of other innovative agents and targets have been tested only in vitro and in the animal model. Thus, more extensive research is mandatory to assess their efficacy and tolerability. In particular, only a minority of these drugs seems suitable for future investigations, considering the cornerstones of the endometriosis management, as well as possible side-effects and impact on quality of life.
